# Ultrasonographic findings in cows with left displacement of abomasum, before and after reposition surgery

**DOI:** 10.1186/s12917-018-1358-7

**Published:** 2018-02-12

**Authors:** Xin-Wei Li, Qiu-Shi Xu, Ren-He Zhang, Wei Yang, Yu Li, Yu-Ming Zhang, Yu Tian, Min Zhang, Zhe Wang, Guo-wen Liu, Cheng Xia, Xiao-Bing Li

**Affiliations:** 1 Key Laboratory of Zoonosis, Ministry of Education, College of Veterinary Medicine, Jilin University, 5333 Xi’an Road, Changchun, Jilin 130062 China; 2 College of animal science and veterinary medicine, Heilongjiang Bayi Agricultural University, Daqing, 163319 Heilongjiang China; 3 College of Animal Science and Technology, Anhui Agricultural University, 130 West Changjiang Road, Hefei, 230036 Anhui China

**Keywords:** Cows, Diagnosis, Left displacement of abomasum, Ultrasonography

## Abstract

**Background:**

The natural incidence of left displacement of abomasum (LDA) in dairy cows was high. The diagnosis of LDA usually relies on characteristic physical exam findings but that transabdominal ultrasound is a useful technique that has been applied to the diagnosis of gastrointestinal diseases of dairy cows in equivocal cases.

**Methods:**

Forty dairy cows with LDA were clinically and ultrasonographically examined to determine the position and the echogenic property of the abomasum. The cows were examined ultrasonographically on the left side, from the 9th intercostal space (ICS) to the 12th ICS as well as the ventral left abdomen before and after reposition surgery.

**Results:**

The vital signs were within normal range in most of the cows and the ‘pinging’ were clearly heard in 37 cows. The abomasal gas cap was visualized from the 9th to 12th ICS in 37 cows and characterized by reverberation artifacts. The abomasal ingesta appeared as homogeneous hypoechoic fluid with scattered hyperechoic foci and were mainly visible in the median region and ventral region of the 9th to 11th ICS in 35 cows. The pyloric canal was detected from the ventral left abdomen wall in 30 cows and appeared as a loop with hypoechogenic wall and echogenic luminal contents in cross section.

**Conclusion:**

These typical ultrasonograms, including reverberation artifacts, homogenous hypoechoic structures, are important diagnostic feature in ultrasonography of LDA. Furthermore, the circular acoustic image structure of the pyloric canal is an important characteristic of LDA, so it can be used as an important diagnostic basis of LDA.

## Background

Since the first case was reported in 1950s [1], left displacement of abomasum (LDA) has become an important disease in dairy cows. LDA is a multifactorial disease that the abomasum partially or completely displaces between the left abdominal wall and rumen. Reduction of milk yield and risk of adhesions make it necessary to diagnose LDA early and precisely. Auscultation and simultaneous percussion or ballottement on the left mid-flank area is a traditional diagnostic method. In most LDA cases, an area of high-pitched ‘pinging’ will be heard. Although a diagnosis usually can be made directly by the specific ‘pinging’, some intensive methods such as rectal examination, blowing air into the rumen through a stomach tube or abomasocentesis are required to differentiate rumen collapse syndrome, rumen tympany and peritonitis pneumoperitoneum from LDA [2]. Furthermore, laparoscopic exploratory, an invasive procedure, is also recommended to make definite diagnosis in borderline cases [3].

In recent times, the use of transabdominal ultrasound has become more widespread over the past 20 years to diagnosed abdominal disorders in cattle [4, 5]. Some studies about ultrasonographic findings of abomasum in healthy cows or LDA cows have been published [6, 7]. According to these studies, the abomasum can be seen well through ultrasonography in both normal and displaced state. These studies indicated that ultrasoundis a helpful tool to diagnose the LDA. However, the interpreting of the ultrasonogram is still not easy to undertake. The present study was designed to ultrasonographically examine the left abdomen of LDA cows before and after reposition surgery, in order to obtain more useful differentiating features of LDA.

## Methods

### Animals

Forty Holstein cows (2–7 years old; 5 ± 1.6 years) were selected by experienced veterinarians depending on auscultations and percussion in a 10,000-cow dairy farm in Changchun, China. All these cows were finally confirmed to be LDA via exploratory laparotomy and underwent an abomasum reposition surgery. The study protocol was approved by the Ethics Committee on the Use and Care of Animals, Jilin University (Changchun, China). During the experimental work, the cows were housed in a climate-controlled barn in individual tie stalls to reduce environmental effects.

### Clinical examination

Simultaneous auscultation and percussion were performed over the left flank of the cows as Richmond described [8]. The heart rate, respiratory rate and rectal temperature of these cows were recorded.

### Ultrasonographic examination

**Fig. 1:**
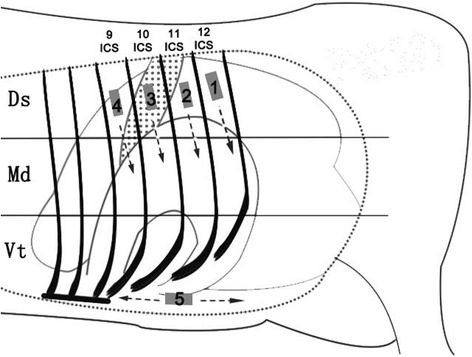
Examination routes. Routes 1–5: Ultrasonographic routes which the transducer followed systematically. The rib cage was divided into three regions: Dorsal (Ds), Median (Md), and Ventral (Vt). The presence of abomasal ultrasonographic characteristics in each region was respectively noted. ICS: intercostal space

The ultrasonographic examination was performed using a lightweight real-time B-mode scanner with a 3.5 MHz low-frequency curvilinear transducer (Shantou Institute of Ultrasonic Instruments Co., Ltd., Shantou, China; Model: CTS-7700V). The penetration depth of transducer was 15 cm. The procedure was performed according to the study of Braun et al., [6]. The cows were unsedated and standing during the examination. The hair was clipped over the left 9~12th intercostal spaces and the left ventral abdominal wall. After the application of transmission gel (ULT0250, Health & Beyond), these intercostal space was scanned, beginning dorsally and progressing ventrally with the transducer held parallel to the ribs (Fig. 1). The ventral left abdomen was scanned with the transducer held parallel to the long axis of the cows (Fig. 1). The 9~12th intercostal spaces were divided into three regions: Dorsal (Ds), Median (Md), and Ventral (Vt) (Fig. 1), and in each region the presence of abomasal characteristics such as abomasal ingesta, abomasal gas cap, and abomasal folds was noted. The same area was examined again immediately after reposition surgery of abomasum.

### Treatment

A right flank laparotomy was performed in all the cows to confirm the diagnosis and then a right flank pyloropexy was adopted to immobilize the abomasum (Mueller [2]).

## Results

### Clinical findings

The vital signs were within normal range in the cows, except for five cows with concurrent disease (two with endometritis, three with mastitis). The heart rate was 64 to 110 (81 ± 12) beats/min, the respiratory rate was 15 to 50 (23 ± 8) breaths/min and the rectal temperature ranged from 38.5 to 40.1 (38.8 ± 0.3). Simultaneous auscultation and percussion over the left flank revealed different degrees of ‘pinging’ in the cows. In 24 cows, the ‘pinging’ could be heard distinctly on the dorsal third of the 11th, 12th and 13th rib. In 13 cows, the ‘pinging’ could be heard only as high as the middle third of the rib cage. Three of the cows had only vague or intermittent ‘pinging’ to be heard.

### Ultrasonographic findings

**Fig. 2:**
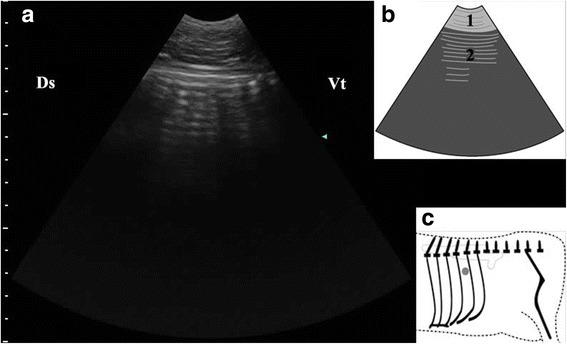
Ultrasonogram of abomasal gas cap (**a**), schematic diagram (**b**) and position of transducer (**c**). A 3.5 MHz low-frequency curvilinear transducer was placed in the left dorsal part of 11th ICS of a cow with LDA, some reverberation artifacts produced by abomasal gas cap appeared as parallel lines. 1-thoracic wall; 2-Reverberation artifacts; Ds-dorsal; Vt-ventral; Grey spot-position of transducer

Before the reposition surgery, the abomasum could be visualized in all of the left 9th, 10th, 11th, 12th intercostal space (ICS) of 37 cows, and could be only identified in the left ventral abdomen region in three cows. The ultrasonographic findings were similar in nearly all cows with LDA consisting of a dorsal gas cap, echogenic luminal ingesta and visualization of the pyloric canal. (Table 1). Reverberation artifact was observed while the transducer was put on the dorsal part of the examined ICSs in all the cows (Fig. 2).

**Table 1:** Ultrasonographic findings of 40 cows with LDA

Exploratory area	Findings	Number of animals
Dorsal part of rib cage	Reverberation artifacts	40
Median part of rib cage	Reverberation artifacts	37
	Homogenous hypoechoic structure	23
Ventral part of rib cage	Reverberation artifacts	14
	Homogenous hypoechoic structure	32
	Heterogeneous echogenic structure	5
	Wavy or sickle shaped echogenic strips	26
Left ventral abdomen region	Homogenous hypoechoic structure	3
	Wavy or sickle shaped echogenic strips	2
	Echogenic loop	30

**Fig. 3:**
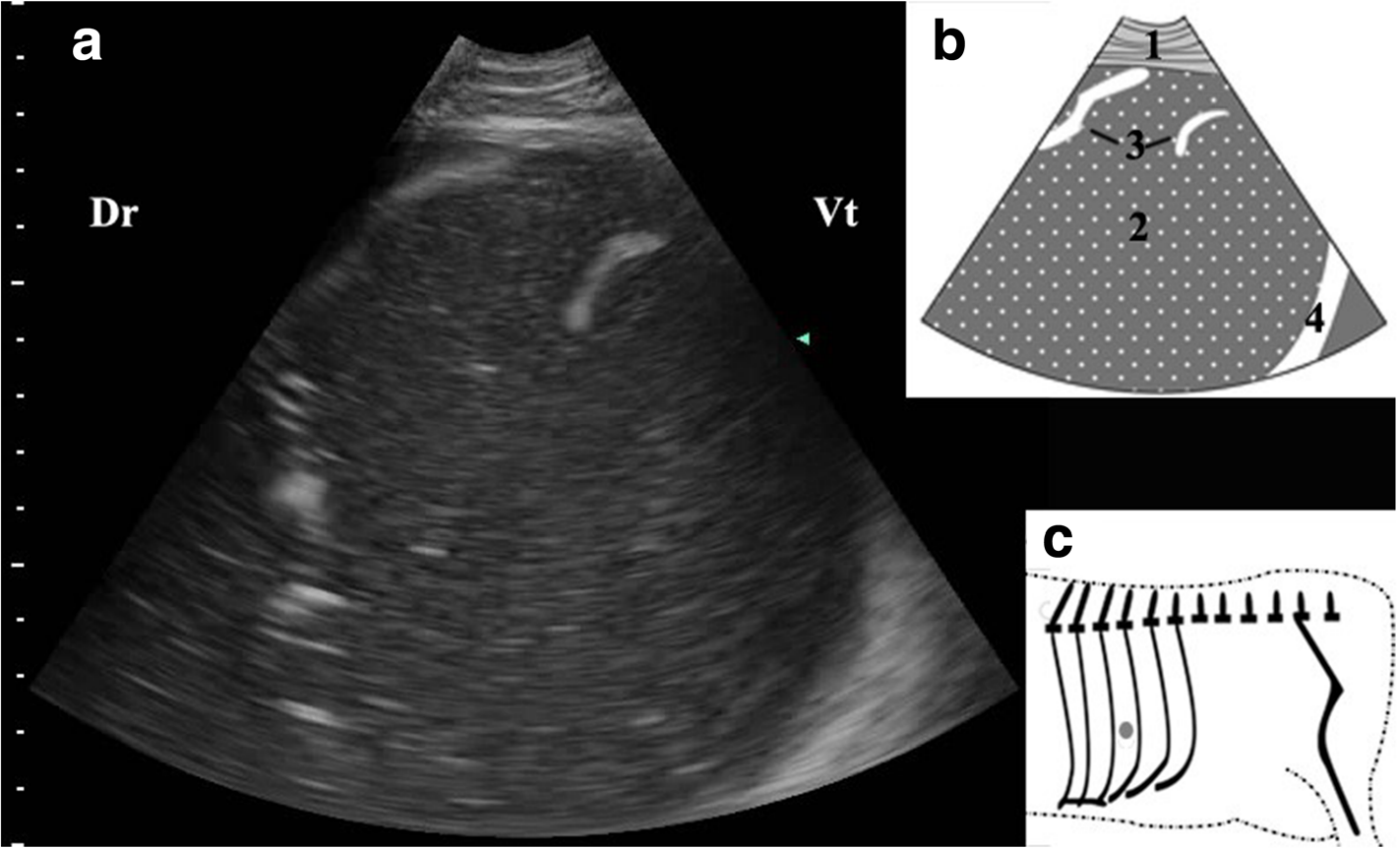
Ultrasonogram of abomasal ingesta (**a**), schematic diagram (**b**) and position of transducer (**c**). A 3.5 MHz low-frequency curvilinear transducer was placed on the ventral part in 10th ICS on the left side of a cow with LDA, homogenous hypoechoic structures with echogenic spots produced by abomasal ingesta appeared. 1-thoracic wall; 2-abomasal ingesta; 3-abomasal fold; 4-ruminal wall; Ds-dorsal; Vt-ventral; Grey spot-position of transducer

**Fig. 4:**
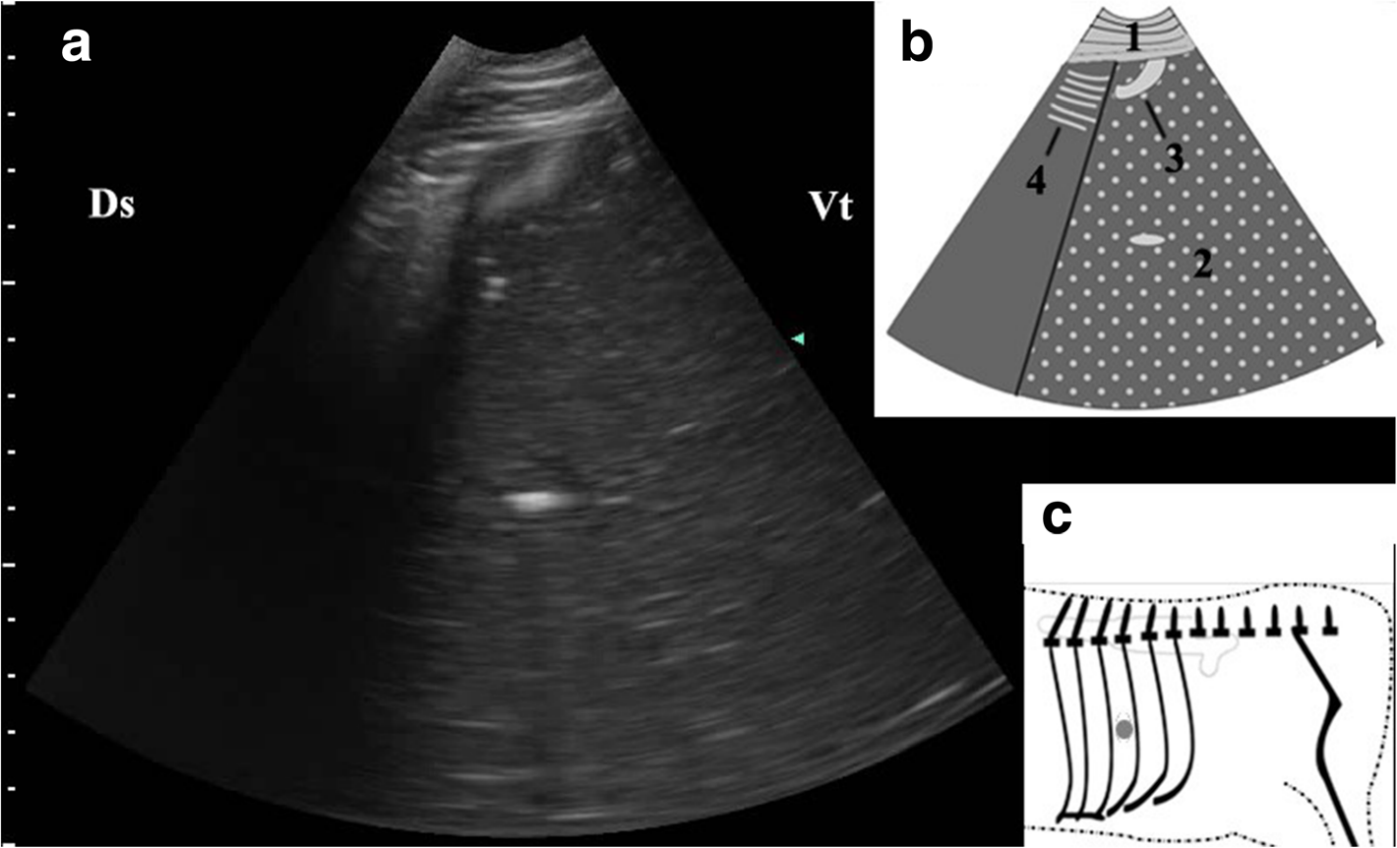
Ultrasonogram of interface between abomasal gas and ingesta (**a**), schematic diagram (**b**) and position of transducer (**c**). A 3.5 MHz low-frequency curvilinear transducer was placed in the median part of 10th ICS on the left side of a cow with LDA, an interface between the gas cap and the ingesta appeared. 1-thoracic wall; 2-abomasal ingesta; 3-abomasal fold; 4-Reverberation artifacts; Ds-dorsal; Vt-ventral; Grey spot-position of transducer

**Fig. 5:**
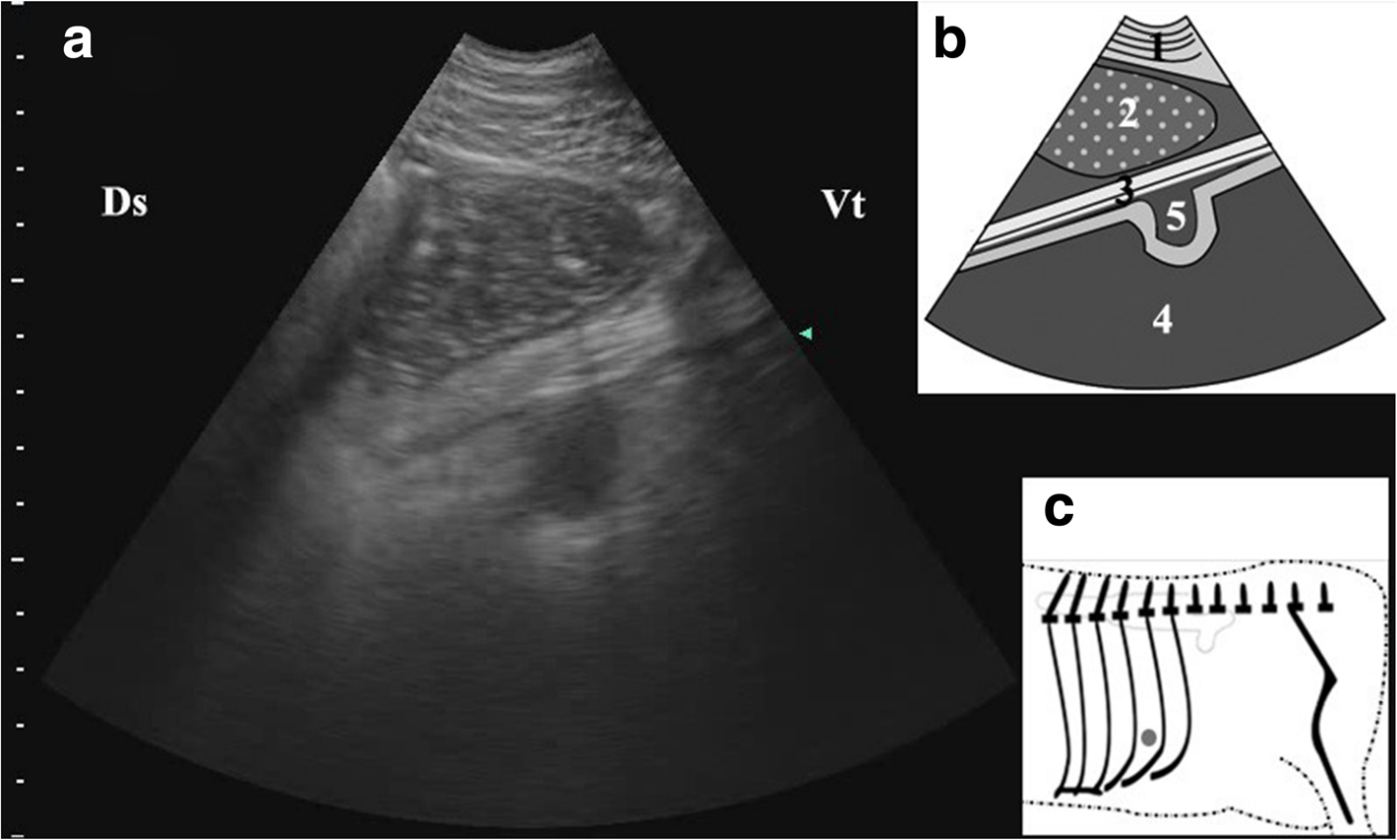
Ultrasonogram of the left longitudinal groove of the rumen median to abomasum (**a**), schematic diagram (**b**) and position of transducer (**c**). A 3.5 MHz low-frequency curvilinear transducer was placed in the ventral part of 11th ICS on the left side of a cow with LDA, the left longitudinal groove of the rumen appeared as a typical notch median to the abomasum. 1-thoracic wall; 2-abomasal ingesta; 3-greater omentum; 4-rumen; 5-left longitudinal groove of the rumen; Ds-dorsal; Vt-ventral; Grey spot-position of transducer

Abomasal ingesta came into view, while the transducer was placed on the median or ventral part of the ICSs. These ingesta were visualized from the 9th to the 11th ICS of 29 cows and in 9th–12th ICS of eight cows. Abomasal ingesta mostly appeared as homogenous hypoechoic structures with echogenic spots (Fig. 3); however there were some cows (*n* = 5) whose abomasal ingesta appeared messy (Fig. 3). There was an interface between the gas cap and the ingesta beneath which reverberation artifacts disappeared abruptly and the echogenic ingesta appeared clearly (Fig. 4). The abomasal ingesta depth was usually beyond the maximal penetration depth (15 cm) of the transducer at start (Fig. 4) and only when the transducer moved ventrally, the ruminal wall which immediately contacted with the left abdominal wall in healthy cows could be visible medial to the abomasum as a thick, smooth echogenic band (Fig. 3). The left longitudinal groove of the rumen was identified medial to the abomasum as a typical notch in ventral part of 11th intercostal space in one cow (Fig. 5). Some abomasal folds were usually observed within the abomasal ingesta as wavy or sickle shaped echogenic strips in 30 cows (Fig. 3).

**Fig. 6:**
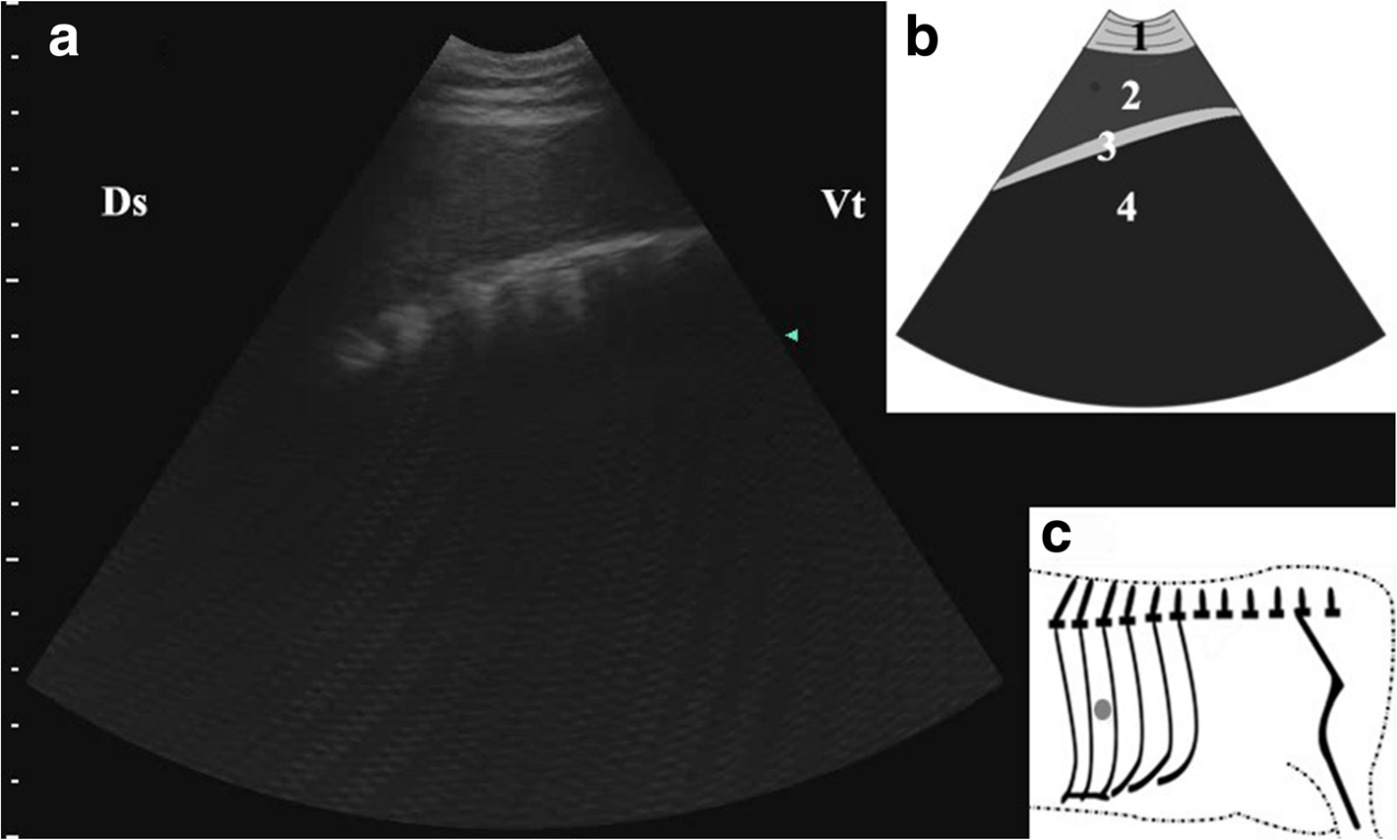
Ultrasonogram of spleen (**a**), schematic diagram (**b**) and position of transducer (**c**). A 3.5 MHz low-frequency curvilinear transducer was placed in the median part of 9th ICS on the left side of a cow just after abomasum reposition surgery, the spleen appeared as homogenous hypoechoic structures. 1-thoracic wall; 2-spleen; 3-ruminal wall; 4-rumen; Ds-dorsal; Vt-ventral; Grey spot-position of transducer

After the reposition surgery, the abomasal characteristic findings mentioned above left no trace in the same exploring area in all the cows, except that some reverberation artifacts was still observed in the dorsal region of the ICSs (Fig. 6). The ultrasound examination of the left abdomen became uncomplicated and the rumen and spleen returned to their normal position.

### Exploratory laparotomy findings

The diagnosis of LDA was confirmed in all the cows via exporatory laparotomy. The abomasum could be felt reclining on the dorsal rumen and distended in 37 of the cows, when the hand advanced over the dorsal rumen across to the dorsal left side of the abdomen. In the other three cows, the abomasum could be palpated only when the hand went under the rumen across to the ventral left side of the abdomen.

## Discussion

In dairy farms, diagnostic ultrasound is mainly used to the diagnosis and management of reproductive conditions. However, more and more studies have indicated that ultrasonography is useful to the diagnosis of some nonreproductive diseases of cows, especial gastrointestinal diseases [8]. In the present study, we showed that the abomasum of the cows with LDA could be generally ultrasonographically identified in the left abdominal cavity. The ultrasonograms probing from the left 9th to 12th ICS and the left ventral abdomen were considerably different before and after reposition surgery in the cows with LDA.

In healthy adult dairy cows, the abomasum could be imaged in ventral abdominal region approximately 8–15 cm caudal to the xiphoid process from the ventral midline, and that more of the abomasum was situated to the right of the ventral midline than to the left. The position of the abomasum depends on the degree of ruminal filling and the stage of pregnancy. Abomasal dimensions, position, and volume change markedly during the last 3 months of gestation and first 3 months of lactation [11]. In cows with LDA, because of gases accumulation, the abomasum enlarges and slides leftwards beneath the ruminal atrium and ventralruminal sac, ultimately rising (like a balloon) between the rumen and the abdominal wall [9]. The abomasal gas cap as a trait of the distended abomasum can produce reverberation artifacts owing to the reflection of the ultrasound beam backwards and forwards between the transducer and the highly reflective surface of gas-filled abomasum [10]. In the present study, reverberation artifacts appeared in most regions of the 9th to 12th ICS in 37 cows with LDA. However, it disappeared largely after the reposition surgery and only a little remained in the dorsal region. According to the previous studies [11–13], the remaining artifacts in the dorsal region resulted from the exploratory laparotomy obscured examination of the dorsal abdomen for at least 24 h after surgery.

Homogenous hypoechoic structures with echogenic spots could be viewed from the ventral or median part of the left 9th to the 12th ICS in most of the cows in the present study. As stated by previous studies [6, 7, 14], these structures were produced by the abomasal ingesta and could not be viewed from the same region in normal cows or other diseased cows. The vanishment of these structures in the cows, after the reposition surgery, further clarified this conclusion. The abomasal folds were recognized in 28 (70%) of the cows with LDA. In contrast with healthy cows, cows with displaced abomasum have more chances to show the abomasl folds [14].

Earlier research about the ultrasonography of LDA mainly described the findings probed from the left 10th to the 12th ICS [6, 7, 11]. In the present study, we extended the examining area to the ventral left abdomen and some interesting ultrasonograms were found. The pyloric canal was visualized from the ventral left abdomen in 30 (75%) of the cows with LDA. Ultrasonographic description related to LDA about pyloric canal in cows is rare and one study reported that the pylorus was positively visible from the right 10th ICS in one healthy cow [6]. The pyloric canal, as part of the abomasum, has a unique circular structure in the ultrasonography. However, the ultrasonographic image of pyloric canal is often confused with small intestine, so it is difficult to observe in normal cattle. Our findings could be explained by that when the body of the abomasum displaced to the left abdomen and trapped, the pylorus canal was also dragged to the left abdomen cavity but placed more ventrally as the result described in the study of Itoh et al. [15]. The pylorus canal is an important characteristic of LDA, so it is worthy to put some attention on the left ventral abdomen when one proposes to examine a suspected cow ultrasonographically. The characteristic circular acoustic image structure of the pyloric cross section is easier to be identified than the ultrasonographic ingesta, so it can be used as an important diagnostic basis of LDA.

It is enough to make a diagnosis, depending on the findings mentioned above; however things are not always consistent. Just as the typical clinical features like ‘pinging’ usually do not exist in the cows with lesser displacement of abomasum, these ultrasonographic features of abomasum may not be detected in mild cases. In the present study, there were three cows whose abomasum could only be visualized from the ventral left abdominal wall and neither the median or ventral reverberation artifacts nor the pyloric canal ultrasonogram could be found; the abomasal ingesta was looming and less deep but more echogenic. The exploratory laparotomy proved that the abomasum of the three cows was lying ventral to the rumen. Since displacement tends to start in the reticulorumen groove, the abomasum of mild cases usually place near the reticulorumen groove [2]. Therefore extending examining region to the area under the left 9th to 10th costal arches is warranted.

## Conclusions

We conclude that ultrasonography is a useful tool for the diagnosis of LDA. The self-controlled method adopted in our study showed that the characteristic findings such as homogenous hypoechoic structures described by previous studies were produced by LDA. In addition, the abnormal position of pyloric canal found in our study is an important diagnostic feature in ultrasonography of LDA. These characteristics make significant difference between LDA cows and healthy cows, so one can depend on them to diagnose LDA easily.

## Abbreviations

ICS: Intercostal space
LDA: Left displacement of abomasum
